# O7-8 Mediation effect of physical activity and sedentary behavior on the association of gender with quality of life in adolescents

**DOI:** 10.1093/eurpub/ckac094.056

**Published:** 2022-08-29

**Authors:** Abdou Omorou, Karine Legrand, Johanne Langlois, Edith Lecomte, Serge Briançon

**Affiliations:** 1 CHRU de Nancy, Vandoeuvre-Lès-Nancy, France; 2 Nancy Publique Health School, University of Lorraine, Vandoeuvre-Lès-Nancy, France; 3 Cnam, Nancy, France; 4 Nancy Public Health School, University of Lorraine, Vandoeuvre-Lès-Nancy, France

**Keywords:** physical activity, sedentary behaviour, quality of life, sex, mediation

## Abstract

**Background:**

If boys and girls are known to have different levels of quality of life (HRQoL), less is known about behavioural factors such as physical activity (PA), sedentary behaviour (SB) that can explain it. We aimed to analyse the mediation effect of PA and SB on the association of sex and HRQoL among adolescents.

**Methods:**

2448 adolescents (1378, 56.3% of Girls) from the PRALIMAP trial with 2-year follow-up were included. HRQoL (physical, mental, social and general dimension scores; range 0-100), PA (walking, moderate vigorous and total) and SB (sitting time) were assessed using the Duke Health profile and the IPAQ questionnaires, respectively. The 2-year mean PA and SB were calculated and categorized into high or low PA (? 1hour/day) or SB (2 hours/day out of school). We conducted a mediation analysis to investigate the causal mechanism of gender (reference=Girls) on HRQoL through PA and SB by estimating total effect (TE), natural direct effect (NDE), natural indirect effects (NIE) as well as proportion mediated (PM: proportion of sex-HRQoL relationship mediated by PA or SB).

**Results:**

The direct relationship of gender and HRQoL (NDE) was significant whatever the HRQoL dimension. When using vigorous PA as mediator, PM was estimated at 13.7% (TE:11.0 [9.5 to 12.5]; NIE:1.5[0.9 to 2.1]), 19.4% (TE: 12.3 [10.6 to 14.1]; NIE: 2.4 [1.6 to 3.1]), 70.0% (TE:4.6 [3.1 to 6.2] ; NIE: 3.3 [2.5 to 4.0]) and 25.6% (TE: 9.3 [8.1 to 10.5]; NIE:2.4 [1.8 to 2.9]) for physical, mental, social and general HRQoL, respectively. Similar but less important mediation effect was observed for moderate and walking PA. For Total PA, PM was estimated 7.0%, 8.7%, 47.4% and 14.7% for physical, mental, social and general HRQoL while for SB, the mediation was inverse and less important (PM: -1.5%, -1.4%, -2.0% and -1.5% for physical, mental, social and general HRQoL respectively).

**Conclusion:**

HRQoL is significantly higher in boys compared to girls among adolescents and this difference is shown to be partially mediated by differences in PA practice, especially vigorous PA. Additionally to the impact of adolescent PA in preventing non-communicable diseases in adulthood, it can also enhance their well-being.

